# Biological Activity of the Alternative Promoters of the *Dictyostelium discoideum* Adenylyl Cyclase A Gene

**DOI:** 10.1371/journal.pone.0148533

**Published:** 2016-02-03

**Authors:** Javier Rodriguez-Centeno, Leandro Sastre

**Affiliations:** 1 Instituto de Investigaciones Biomédicas CSIC/UAM, C/Arturo Duperier, 4, 28029, Madrid, Spain; 2 CIBERER, Valencia, Spain; MRC Laboratory of Molecular Biology, UNITED KINGDOM

## Abstract

Amoebae of the *Dictyostelium discoideum* species form multicellular fruiting bodies upon starvation. Cyclic adenosine monophosphate (cAMP) is used as intercellular signalling molecule in cell-aggregation, cell differentiation and morphogenesis. This molecule is synthesized by three adenylyl cyclases, one of which, ACA, is required for cell aggregation. The gene coding for ACA (*acaA*) is transcribed from three different promoters that are active at different developmental stages. Promoter 1 is active during cell-aggregation, promoters 2 and 3 are active in prespore and prestalk tip cells at subsequent developmental stages. The biological relevance of *acaA* expression from each of the promoters has been studied in this article. The *acaA* gene was expressed in *acaA*-mutant cells, that do not aggregate, under control of each of the three *acaA* promoters. *acaA* expression under promoter 1 control induced cell aggregation although subsequent development was delayed, very small fruiting bodies were formed and cell differentiation genes were expressed at very low levels. Promoter 2-driven *acaA* expression induced the formation of small aggregates and small fruiting bodies were formed at the same time as in wild-type strains and differentiation genes were also expressed at lower levels. Expression of *acaA* from promoter 3 induced aggregates and fruiting bodies formation and their size and the expression of differentiation genes were more similar to that of wild-type cells. Expression of *acaA* from promoters 1 and 2 in AX4 cells also produced smaller structures. In conclusion, the expression of *acaA* under control of the aggregation-specific Promoter 1 is able to induce cell aggregation in *acaA*-mutant strains. Expression from promoters 2 and 3 also recovered aggregation and development although promoter 3 induced a more complete recovery of fruiting body formation.

## Introduction

The amoeba *Dictyostelium discoideum* has been the subject of numerous studies on cell motility, adhesion, cell differentiation and morphogenesis due to the singularity of its biological cycle (recently reviewed in [1–3]. This organism lives as individual amoebae on forest soils, feeding on bacteria. However, when the food gets exhausted, thousand of amoebae get together and cooperate to form a fruiting body. Multicellular development initiates by the migration of thousands of amoebae towards aggregation centres to form mounds of cells. Cells subsequently initiate alternative differentiation programs, either as prestalk or as prespore cells. Migratory movements and differential adhesion processes result in the location of prestalk cells at the top of the mound, where they protrude as a tip. Subsequently, prestalk cells move towards the substrate, pile up, get elongated and terminally differentiate to form a stalk. During this process prespore cells remain attached to the upper part of the stalk, rising from the substrate to form a globular sorus. Once the culmination process is completed, prespore cells get dehydrated and surrounded by a protein-cellulose coat to form mature spores. Dissemination of the spores facilitates their arrival to a favourable environment where they germinate, reinitiating the biological cycle.

The developmental programme requires cell communication to coordinate cell migration, adhesion and differentiation [3]. One of the more important signalling molecules used by *D*. *discoideum* is cyclic-AMP (cAMP) [4]. This molecule is used both as a secreted inter-cellular signal and as an intra-cellular second messenger involved in signal transduction [2, 5]. The use of cAMP as an extracellular signal was first characterized in the process of cell aggregation [6]. cAMP is secreted from cells at aggregation centres. Neighbouring cells detect the presence of cAMP and respond by moving towards the increasing cAMP concentration and also by secreting more cAMP, contributing to spread the aggregation signal. This process is periodically repeated and generates cAMP concentration waves that induce the movement of the cells towards aggregation centres [7]. In addition, cAMP as acts as an intracellular signalling molecule during this process. Binding of cAMP to cAR1 (cAMP receptor 1) activates several signalling pathways. Some of them activate the chemotactic movement of the cells [8]. cAR1 activation also induces the synthesis of cAMP inside the cells activating the cAMP-dependent protein kinase A (PKA) pathway. cAMP is also secreted [9] contributing to reinforce the cAMP waves [7]. Later on, morphogenetic movements inside the mound are controlled by cAMP waves originating from the tip region of the developing structures [10]. A high extracellular cAMP concentration is also required for the differentiation of prespore cells into spores [11, 12]. Finally, high extracellular cAMP levels avoid precocious germination of the spores inside the sorus [13]. Intracellular cAMP signalling through PKA activation is also required for all these processes [5].

cAMP levels are controlled by the developmentally-regulated expression and activity of adenylyl cyclases and phosphodiesterases [14, 15]. Extracellular phosphodiesterases, such as PDE1, participate in the generation of the cAMP waves. Regulation of the activity of intracellular phosphodiesterases, such as RegA, is required for cAMP secretion, aggregation and cell differentiation. Cyclic AMP is synthesized by three different adenylyl cyclases (AC), ACA, ACR and ACG [16]. The cyclase ACA, encoded by the *acaA* gene, is expressed from the first hours of development and is responsible for cAMP synthesis during the aggregation process. Strains that are mutated at *acaA* do not aggregate [17]. In addition, mutated cells do not express developmentally-regulated genes induced by cAMP such as *csaA*, *tgrC1* or *tgrC5*, that code for proteins involved in cell adhesion [18]. ACB is encoded by the *acrA* gene, whose expression is strongly induced in prestalk cells from six hours of development. Strains mutated at this gene do not complete the culmination process [19, 20]. The gene coding for ACG, *acgA*, is induced in prespore cells from 12 hours of development [17]. ACG activation induces prespore differentiation into spores [21]. In addition, ACG activity is regulated by osmotic pressure, which is high in the sorus, and represses spore germination [13]. This role in spore differentiation and germination has been conserved in several Dictyostelia species [22].

The functional data therefore indicate that *acaA* is the main adenylyl cyclase involved in aggregation. Evolutionary studies are also in agreement with this proposal. Dictyostelia are composed by over one hundred species divided into four groups according to DNA sequence comparisons. Only one of these groups uses cAMP as a signalling molecule during aggregation while the other groups use other chemoattractant molecules such as Glorin, Folic acid or Pterin [23]. However, the regulatory role of cAMP in morphogenesis, spore differentiation and germination, is similar in species from the four groups [4]. These processes seem to be mainly dependent on ACB and ACG, the adenylyl cyclases better conserved among the Dictyostelia. The use of extracellular cAMP as a chemoattractant seems to be a evolutionary acquisition of group four Dictyostelia species [2]. Oscillatory cAMP secretion during aggregation is regulated by the cAMP receptor cAR1 and proteins involved in cAMP synthesis, degradation and intracellular signalling such as ACA and the extracellular phosphodiesterase PdsA [7]. The genes coding for these proteins have in common their expression from different alternative promoters. The more distal promoters are only present in group-four Dictyostelia and drive gene expression at aggregation while more proximal promoters regulate expression at later developmental stages [24–26]. This observation suggests that the capacity to express these genes during aggregation was obtained by adding distal promoter regions [27].

Analyses of the *acaA* gene promoter region identified the existence of three promoters [26]. The one more distal to the coding region directs *acaA* expression during aggregation, as already mentioned. The intermediate promoter direct *acaA* expression in cells of the prespore region and the more proximal one in prestalk cells at the tip region. The expression of *acaA* in specific regions during post-aggregative development indicates that ACA could play a role in cell differentiation and/or morphogenesis. The identification of the three *acaA* promoters opens the way to study their developmental functions Complementation studies of *acaA* mutant strains using the three promoters to drive expression of the gene are, therefore, reported in this article. Expression of *acaA* from the distal, aggregation-specific, promoter is sufficient for aggregation. However, the developmental process also requires *acaA* expression from promoter 3 for complete recovery of fruiting body morphology and patterns of gene expression.

## Materials and Methods

### Cell culture and transformation

*D*. *discoideum* cells were grown axenically in HL5 media. Cells were alternatively grown feeding on *Klebsiella aerogenes* over SM-agar plates. Cells were transformed by electroporation as previously described [28]. Transformed cells were selected by culture in HL5 media in the presence of 5 μg/ml blasticidin or 10 μg/ml geneticin (G418).

### Multicellular development

Development into fruiting bodies was induced by centrifugation of the cells and spreading of 2x10^7^ cells resuspended in PDF buffer (20 mM KCl, 9 mM K_2_HPO_4_, 13 mM KH_2_PO_4_, 1 mM CaCl_2_, 1 mM MgSO_4_, pH 6.4) on 37-mm Nitrocellulose filters supported by pads saturated in buffer. Cell aggregation was also observed by incubating 10^6^ cells in 2 ml of PDF Buffer in 37-mm cell-culture dishes.

### RNA extraction, Reverse transcription and quantitative PCR

*D*. *discoideum* developing structures were obtained by spreading 2x10^7^ cells on Nitrocellulose filters soaked on PDF buffer. Cells and structures were collected at the indicated times and the RNA extracted using 1 ml of the TRI Reagent (Sigma-Aldrich Inc., St. Louis, MO, USA) according to the manufacturer’s instructions. Isolated RNAs were incubated with DNase I and further purified using the RNeasy Mini Kit (Qiagen, Valencia, CA, USA). Complementary DNAs were generated from 2 μg of RNA using random hexanucleotides as primers (Promega Co., Madison, WI, USA) and the M-MLV reverse transcriptase (Promega Co., Madison, WI, USA). Quantitative PCR reactions were carried on the Step One Real-Time PCR System (Life Technologies, Co. Applied Biosystems, Carlsbad, CA, USA). The Power SYBR^®^ Green PCR Mix (Applied Biosystems) was used for these reactions. The conditions used for the PCR reactions have been described previously [29]. The comparative threshold cycle method [30] was used to determine relative gene expression levels using the large mitochondrial rRNA as endogenous control. The oligonucleotides used as primers are indicated in S1 Table.

### Generation of *acaA*-mutant AX4 strains

*D*. *discoideum* AX4 cells were transfected with a plasmid vector designed to interrupt the *acaA* gene by homologous recombination, kindly provided by Zhi-Hui Chen and Pauline Schaap. Mutant strains were isolated by culture with 5 μg/ml blasticidin and the existence of homologous recombination tested by PCR reactions. Mutant strains did not form aggregates when cultured on *K*. *aerogenes*.

### Generation of plasmid vectors

The plasmid vector pDneo2a-6xMYC [31] was used for *acaA* expression. The XbaI-PstI DNA fragment of the pDneo2a-6xMYC vector containing the Actin 6 promoter was replaced by each of the three *acaA* promoters, obtained by PCR reactions. The oligonucleotides used are shown in S1 Table. The *acaA* protein-coding region was subsequently cloned in the vectors that contained the *acaA* promoters using the PstI and BamHI restriction sites. These restriction sites were incorporated to the *acaA* coding region by PCR using the oligonucleotides shown in S1 Table.

## Results

**Fig 1:**
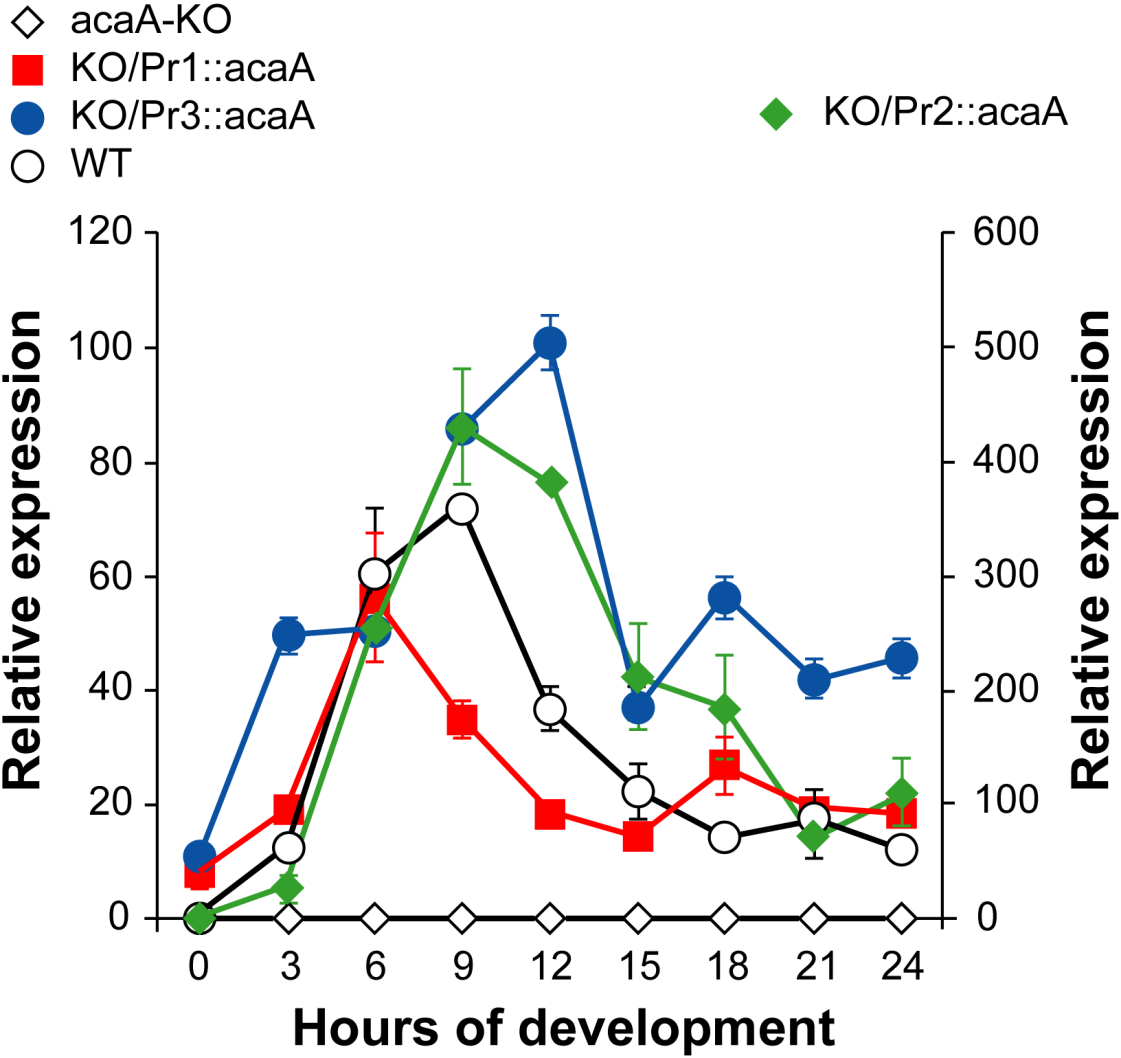
Expression of *acaA* mRNA in Wild-type, *acaA*-mutant and complemented strains. Multicellular development of wild-type AX4 cells (WT), AX4 *acaA* mutant cells (*acaA-*KO) and mutant cells transformed with vectors that express *acaA* from the *acaA* promoters 1 (KO/Pr1::*acaA*), 2 (KO/Pr2::*acaA*) or 3 (KO/Pr3::*acaA*) was induced by spreading 2x10^7^ cells on Nitrocellulose filters. Cells were collected before spreading (time 0) or every three hours of development (3, 6, 9, 12, 15, 18, 21 and 24 hours). RNA was extracted and the levels of *acaA* mRNA determined by reverse transcription and quantitative PCR. The mitochondrial large ribosomal RNA was used as internal control. Relative expression levels are expressed in relation of those of AX4 undiferentiated cells (WT. time 0) for all strain. The left Y axis indicates WT, *acaA*-KO, KO/Pr1::*acaA* and KO/Pr3::*acaA* expression levels and the right Y axis KO/Pr2::*acaA* expression levels. Average values and standard deviations of three experiments made in triplicate are represented.

The *acaA* gene was interrupted by homologous recombination in the *D*. *discoideum* AX4 strain, used for the previous characterization of the *acaA* promoters (S1 Fig), Themutant strain 1 was subsequently transfected with plasmid vectors that express *acaA* under transcriptional control of the three *acaA* promoters. The relative levels of *acaA* mRNA expressed at different developmental stages were determined by RT-qPCR, as shown in Fig 1. The data obtained are in good agreement with those previously reported for the activity of the promoters using short-lived *lacZ*-expression vectors, with the exception of Promoter 3 [26]. The aggregation-specific Promoter 1 is the first one to be activated with maximal expression at 6 hours of development. Promoter 2 showed maximal activity between 9 and 12 hours of development and is the strongest promoter region with about five times more activity than promoters 1 and 3 (right Y-axis in Fig 1). Promoter 3 is active from 3 hours of development showing maximal expression at 12 hours and maintaining about 50% of maximal expression at later developmental stages. However, reporter vector studies showed that this promoter was activated only after 10 hours of development in wild-type AX4 cells, as will be discussed later. Expression from the three *acaA* promoters in the mutant strain was compared with the endogenous *acaA* mRNA expression from the Wild-type (WT) and *acaA*-KO strains (Fig 1). The *acaA* KO strain showed background expression levels throughout development while the WT strain showed the described pattern of *acaA* expression that is maximal between 6 and 9 hours of development. The expression levels of the *acaA* mRNAs from the complemented strains shown in Fig 1 were made relative to the WT *acaA* expression in vegetative cells (0 hours of development) to compare relative levels of expression. The data obtained indicate that promoters 1 and 3 induced levels of *acaA* expression close to those of the WT strain, 60–100 times of maximal induction in the complemented strains compared to 70 times in the WT. In contrast expression levels driven by promoter 2 were about 5 times higher than the WT ones, as mentioned above in the comparison to promoters 1 and 3.

**Fig 2:**
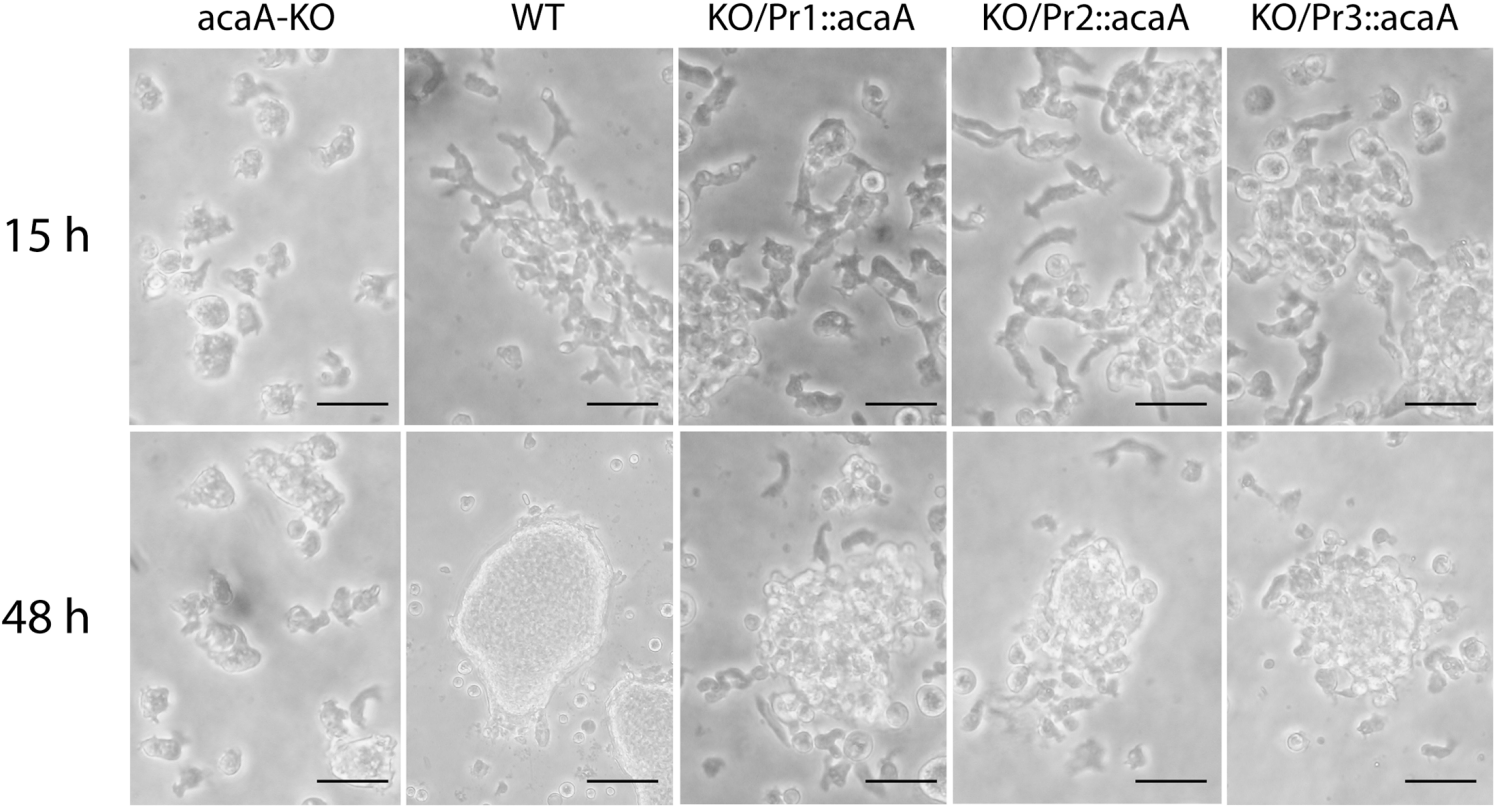
Development of complemented strains under submerged conditions of culture. Wild-type cells (WT), *acaA*-mutant (*acaA*-KO) or *acaA*-mutant cells transfected with *acaA*-expression plasmids vectors under control of the *acaA* promoters 1 (KO/Pr1::*acaA*), 2 (KO/Pr2::*acaA*) or 3 (KO/Pr3::*acaA*) were suspended in Phosphate-based PDF buffer and cultured on plastic dishes. Cell morphology and aggregation were observed after 15 (panel A) or 48 (panel B) hours of culture. Scale bar: 10μm.

It has been described that *acaA* mutant strains do not aggregate because of the absence of synthesis of the cAMP required for chemotaxis. It is expected that *acaA* expression in the KO strain would recover aggregation and/or subsequent developmental processes. The formation of cell aggregates was first studied by starvation of cells submerged in buffered solutions. The *acaA*-KO AX4 cells did not aggregate even after 48 hours of incubation (Fig 2). Mutant cells expressing the Pr1::*acaA* construct were polarized and migrated to form cell aggregates as wild-type cells although the aggregates formed were looser and tight aggregates were not observed after 48 hours of incubation (Fig 2A). Mutant cells expressing Pr2::*acaA* and Pr3::*acaA* constructs were also polarized by 15 hours of starvation (Fig 2) and formed tight aggregates by 48 hours (Fig 2). Cell aggregates were, however, smaller than those formed by the wild-type strain under these conditions.

**Fig 3:**
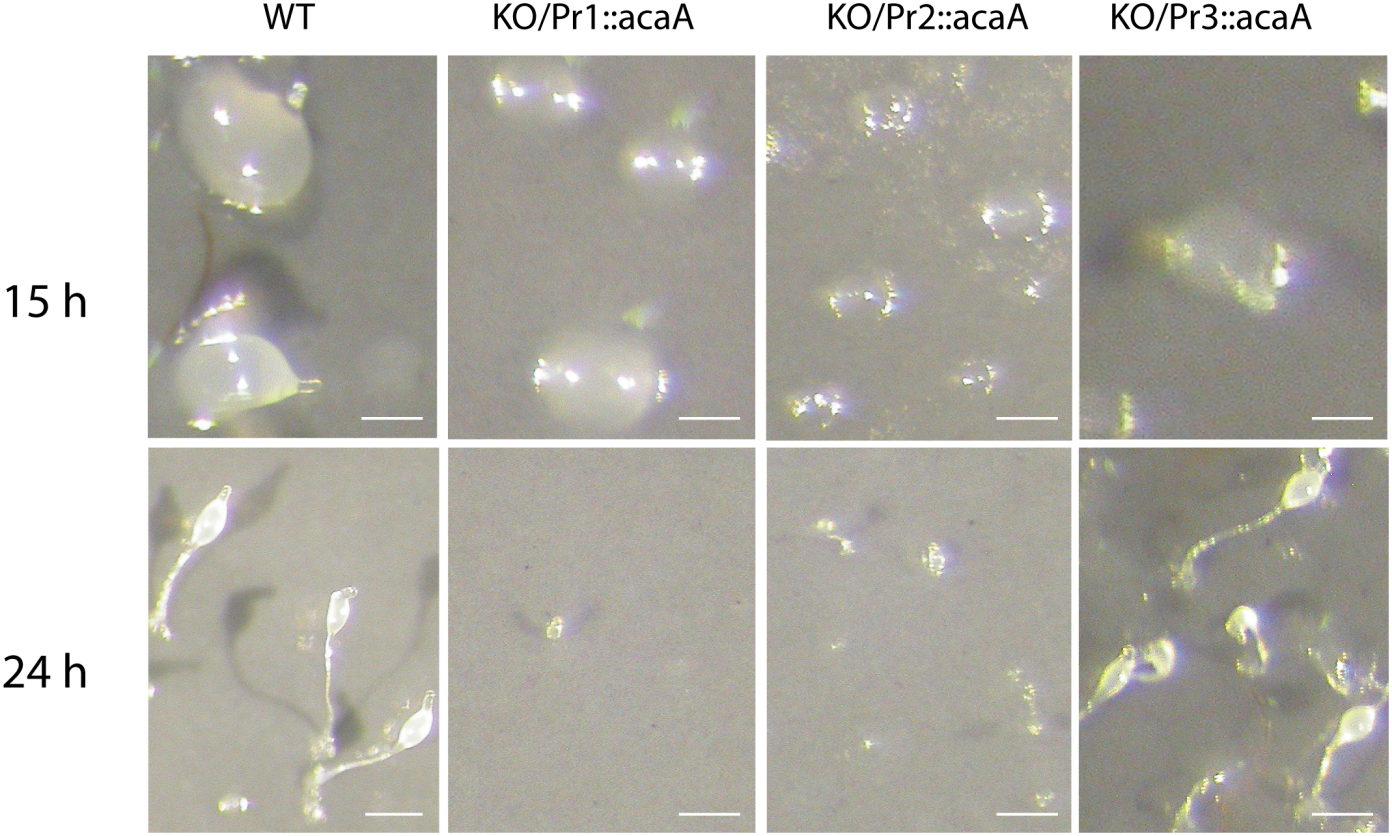
Development of the complemented strains on Nitrocellulose filters. The development of wild-type cells (WT) and *acaA*-mutant cells transfected with *acaA*-expression plasmids under control of the *acaA* promoters 1 (KO/Pr1::*acaA*), 2 (KO/Pr2::*acaA*) or 3 (KO/Pr3::*acaA*) was studied. Cells from the different strains were harvested, suspended on PDF buffer and deposited on Nitrocellulose filters at a concentration of 1.2 Cells/cm^2^. Pictures were taken at 15 and 24 hours of culture. Scale bar: 0.1 mm.

Post-aggregation development was studied by starving the cells on top of Nitrocellulose filters. Wild-type cells formed aggregates by 15 hours of starvation. Aggregated cells then proceeded into a morphogenetic program to form fruiting bodies and culminating structures could be observed by 24 hours of development (Fig 3, WT). Cells where the *acaA* gene had been knocked out did not form aggregates and later developmental structures, as already mentioned. Expression of the Pr1::*acaA* construct in *acaA*-KO cells recovered cell aggregation (Fig 3 KO/Pr1::*acaA*) but posterior development was slowed down. Only a reduced number of very small finger structures and fruiting bodies were formed by 24 hours of development (Fig 3 KO/Pr1::*acaA*). Mutant cells expressing the Pr2::*acaA* construct formed aggregates by 15 hours of development that were smaller than the WT ones. Post-aggregation development was not completely recovered and very few small fruiting bodies were observed 24 hours after starvation (Fig 3 KO/Pr2::*acaA*). Expression of *acaA* under control of Promoter 3 in *acaA*-KO cells almost completely recovered the developmental process (Fig 3 KO/Pr3::*acaA*).

The development of each strain was characterized in more detail by determining the temporal pattern of expression of four genes involved in aggregation, cell adhesion or differentiation. Among these genes, *carA* codes for the cAMP receptor 1 and is expressed in the first stages of the developmental program. This receptor is activated by extracellular cAMP, initiating signalling pathways that induce cAMP synthesis and cell migration. Both activities are required for cell aggregation. The initial induction of *carA* expression is dependent on cell starvation but independent of extracellular cAMP signalling [32]. The second gene analyzed, *tgrC5*, codes for a protein involved in cell adhesion that is required for mound compaction and cell type segregation. In contrast to *carA*, the induction of *tgrC5* is dependent on the previous existence of extracellular cAMP signalling [33].

**Fig 4:**
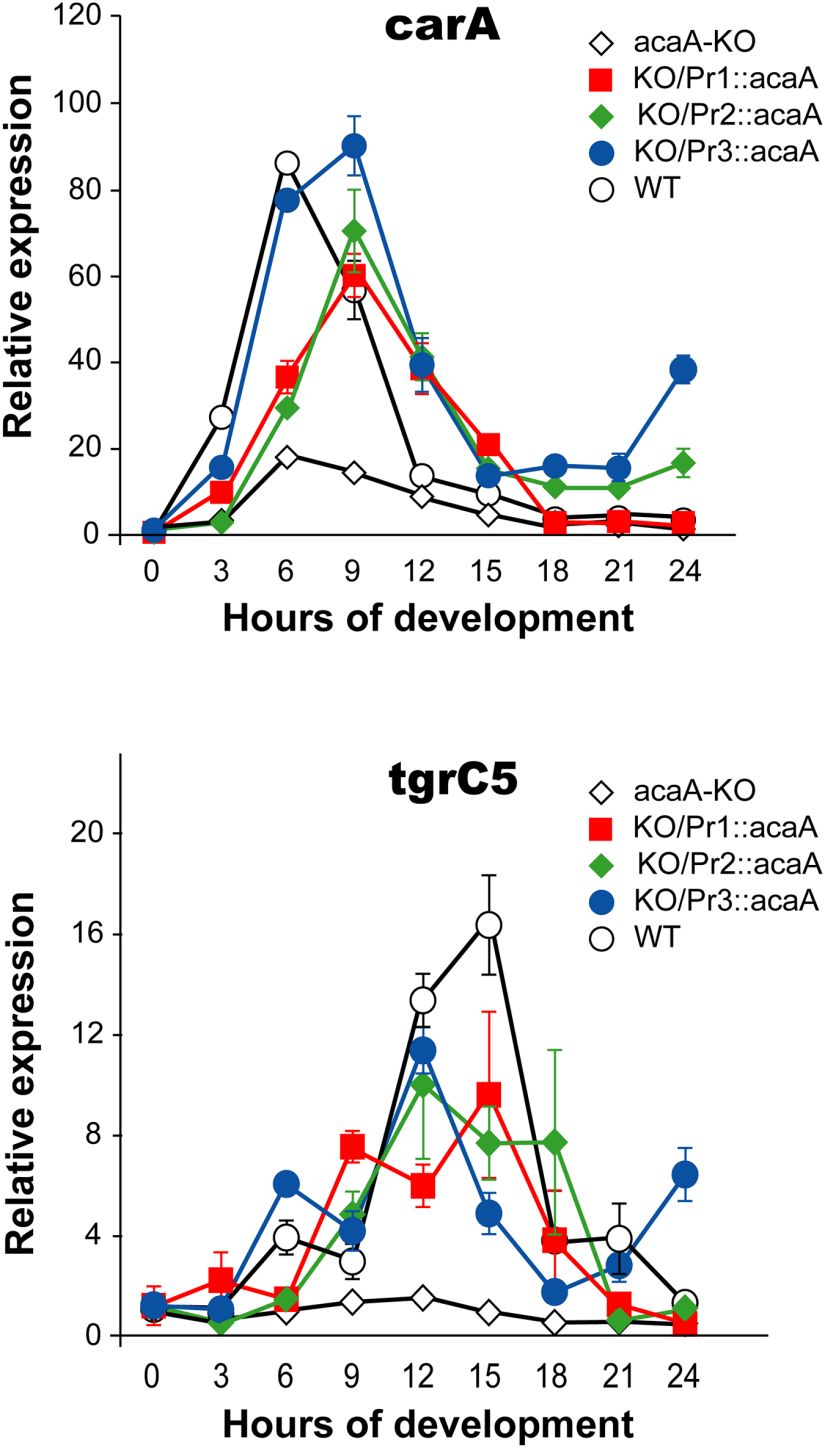
Expression of aggregation-specific genes in complemented *acaA*-mutant strains. Cells from the wild-type AX4 strain (WT), the *acaA*-mutant strain (*acaA*-KO) or the mutant strain transformed with *acaA* expression vectors under control of the *acaA* promoters 1 (KO/Pr1::*acaA*), 2 (KO/Pr2::*acaA*) or 3 (KO/Pr3::*acaA*) were deposited on Nitrocellulose filters soaked on the phosphate-based PDF Buffer to induce development. RNAs were isolated from cells collected either before placing them on the filters (time 0) or at different times of development (3, 6, 9, 12, 15, 18, 21 and 24 hours). The expression of the genes coding for cAMP receptor 1 (*carA*, upper panel) and the cell-adhesion molecule *TgrC5* (*tgrC5*, lower panel) was determined by reverse transcription and quantitative PCR. Expression of the mitochondrial large ribosomal RNA was used as internal control. Expression at each developmental stage was normalized to that of non-starved cells (time 0) for each gene and strain. Average values and standard deviations of three experiments made in triplicate are represented.

The expression pattern of *carA* and *tgrC5* genes during development of WT, *acaA*-KO and KO strains expressing *acaA* under control of the three *acaA* promoters is shown in Fig 4. The expression of *carA* is induced during the first hours of development, with maximal expression at 6–9 hours. As expected, the temporal expression pattern is similar in the WT and *acaA*-KO strains since it is not dependent on cAMP signalling. However, *carA* mRNA levels are lower in the *acaA*-KO strain than in the WT and complemented KO strains indicating stimulation of *carA* expression by cAMP signalling. The expression of *tgrC5* is dependent on cAMP signalling and this gene is not induced in the *acaA* KO strain. Expression of *acaA* under control of any of the promoters resulted in the induction of *tgrC5* expression. The induction was observed at 6 hours of development with maximal levels at 12–15 hours in the WT and complemented strains (Fig 4).

**Fig 5:**
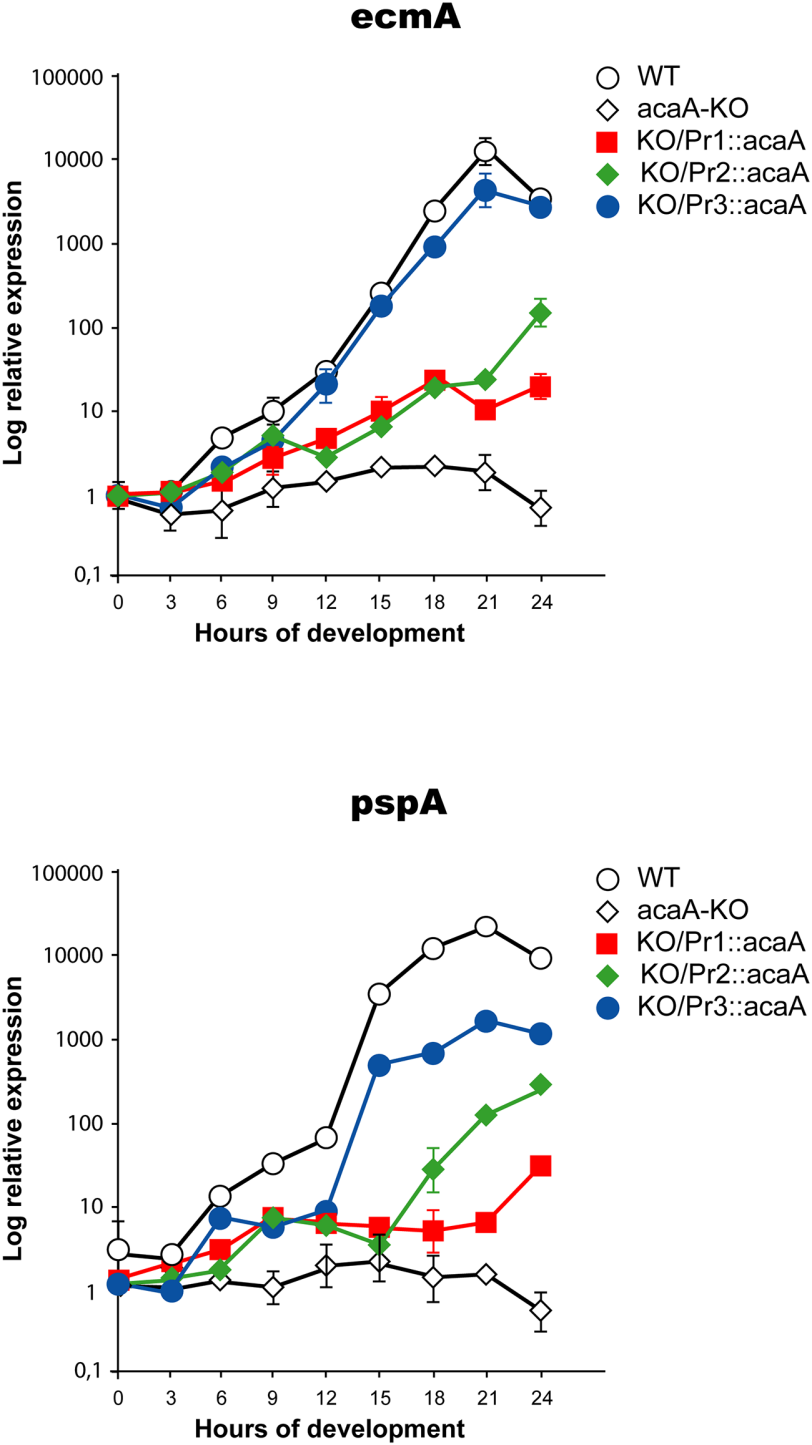
Expression of cell differentiation genes in complemented *acaA*-mutant strains. Cells from the wild-type AX4 strain (WT), the *acaA*-mutant strain (*acaA*-KO) and the mutant strain transfected with vectors that drive *acaA* expression under control of each of the *acaA* promoters 1 (KO/Pr1::*acaA*), 2 (KO/Pr2::*acaA*) or 3 (KO/Pr3::*acaA*) were deposited on PDF-soaked Nitrocellulose filters. Cells were collected either before starvation (time 0) or after several culture periods (3, 6, 9, 12, 15, 18, 21 and 24 hours) and their RNA extracted. The relative expression levels of the prestalk-specific *ecmA* gene (upper panel) and the prespore-specific *pspA* gene (lower panel) were determined by reverse transcription and quantitative PCR. Expression levels were normalized using the mitochondrial large ribosomal RNA expression levels and made relative to the expression observed in non-starved cells (time 0). Results are represented on a logarithmic scale in the Y axis. Average values and standard deviations of three experiments made in triplicate are represented.

The other two genes studied, *ecmA* and *pspA*, are expressed in differentiated prestalk and prespore cells, respectively [34, 35]. Their induction is dependent on the correct fulfilment of previous developmental processes and is used as indicator of developmental progress. The expression patterns observed for the prestalk and prespore genes are shown in Fig 5. None of the genes is expressed in *acaA* KO strain. However, both genes are expressed in the *acaA*-KO strains expressing *acaA* from the three promoters, and in the WT strain, from 15 hours of development onwards. Expression levels were, however, very different (Fig 5). The expression of both genes was induced several thousand times in the WT strain, in comparison to proliferating cells (0 hours of development). Comparable levels of expression were only obtained in the *acaA*-KO strain expressing the Pr3::*acaA* construct. The *acaA*-KO strains expressing Pr1::*acaA* and Pr2::*acaA* showed limited *ecmA* and *pspA* induction, 25–30 and 150–250 times respectively, indicating lower levels of prespore and prestalk cells differentiation.

**Fig 6:**
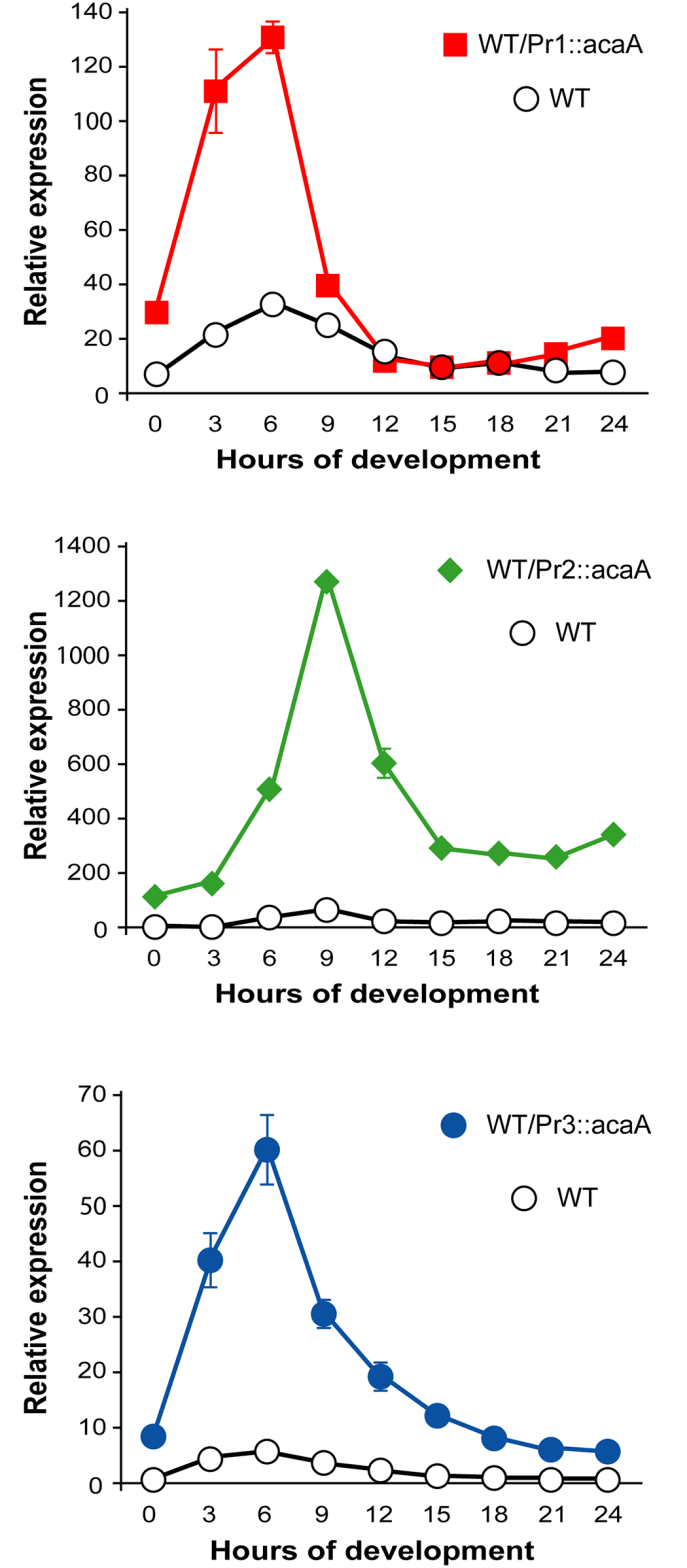
Developmental patterns of *acaA* expression observed in WT strains that express *acaA* under control of the three *acaA* promoters. The plasmids containing the *acaA* gene under control of the three *acaA* promoters were transfected in wild-type AX4 cells. Transfected cells were deposited on Nitrocellulose filters soaked on PDF buffer to induce multicellular development. Cells were collected either before starvation (time 0) or after different times of culture on the filters (3, 6, 9, 12, 15, 18, 21 and 24 hours) and their RNA isolated. Expression levels of the endogenous gene (WT) and the *acaA* mRNA transcribed from the three expression vectors (WT/Pr1::*acaA;* WT/Pr2::*acaA;* WT/Pr3::*acaA*) were determined by reverse transcription and quantitative PCR. Expression patterns of AX4 strains transfected with the Pr1::acaA (upper panel), Pr2::acaA (middle panel) and Pr3..acaA (lower panel) vectors are shown. The mitochondrial large ribosomal RNA was used as internal control and the expression values made relative to those of the endogenous *acaA* mRNA of non-starved cells (WT; time 0) in each panel. Average values and standard deviations of two experiments made in triplicate are represented.

Previous studies had shown that *acaA* over-expression produced small aggregation fields and small fruiting bodies [36] [37]. The *acaA*-KO strain transfected with Pr1::*acaA* and Pr2::*acaA* plasmid vectors resulted in small fruiting bodies and induced *acaA* expression levels higher than those of the wild type strain, as determined by RT-qPCR. We wanted to determine if this high expression levels could have any functional consequences on the developmental process. Therefore, the wild type AX4 strain was transfected with the three acaA expression vectors. Comparative levels of expression of *acaA* mRNAs transcribed from the endogenous gene or from the plasmid vectors were compared by RT-qPCR using specific pairs of oligonucleotides. Reverse primers complementary to the 3’ untranslated region, immediately downstream of the stop codon from the endogenous gene or the expression vector and a common forward primer from the *acaA* coding region (S1 Table) were used in these experiments. The results obtained were made relative to the endogenous *acaA* mRNA expression level in vegetative cells (0 hours) for each strain (Fig 6). The temporal patterns of expression of *acaA* expressed from the plasmid vectors from promoters 1 and 2 were similar to those observed in the *acaA*-KO strain (Fig 1), although not identical. Promoter 1 showed higher expression at 3 hours in the WT strain. The endogenous acaA mRNA showed maximal expression at 6 hours of development although induction levels were lower than those observed in untransfected WT cells, 70 times versus 30 times (compare Figs 1 and 6). Therefore, expression from this promoter preceded the peak of induction of the endogenous gene by 3 hours. Promoter 2 drove maximal expression at 9 hours in WT and *acaA*-KO cells. The temporal pattern of expression was very similar to that of the endogenous gene although the relative expression obtained from the plasmid (1200 times) was much higher than that of the endogenous gene (75 times, Fig 6), in concordance with the data obtained in the *acaA*-KO strain. Promoter 3 induced maximal relative expression at 6 hours of development in the WT strain, which is in contrast with the results obtained in *acaA*-KO cells (maximal induction at 12 hours, Fig 1). Induction of the endogenous *acaA* mRNA is low in the WT/Pr3::acaA strain, 6 times of maximal induction at 6 hours of development, as compared with 70 at untransfected WT cells. These results could indicate a regulatory interaction between Promoter 3 and the endogenous gene that is not observed for promoters 1 and 2, as discussed later.

**Fig 7:**
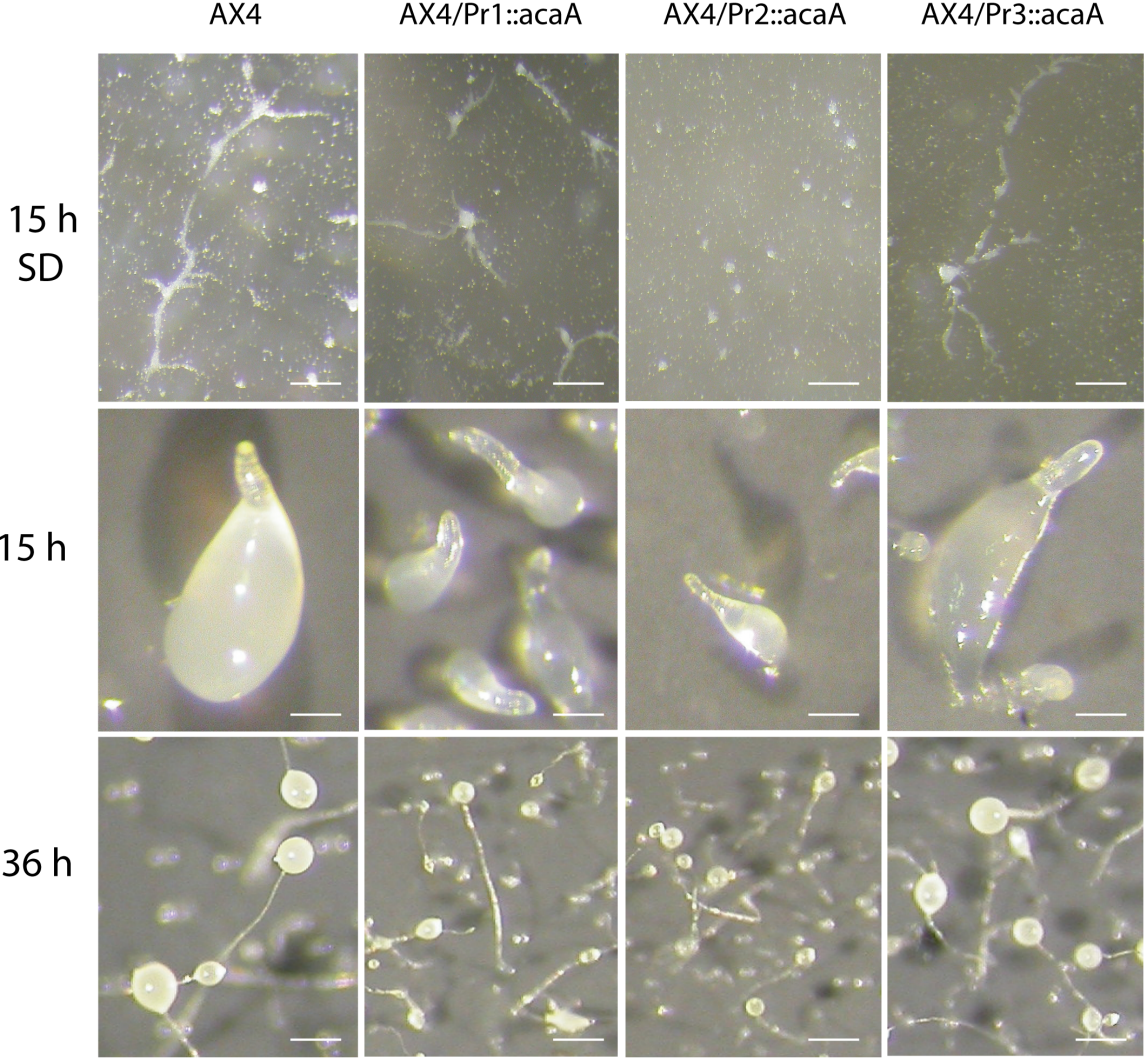
Development of the AX4 cells expressing *acaA* from the three *acaA* promoters. WT Ax4 cells were transfected with the expression vectors driving *acaA* expression from each of the three *acaA* promoters (WT/Pr1::*acaA;* WT/Pr2::*acaA;* WT/Pr3::*acaA*). Upper panels show the aggregation streams formed after development of these cells and the WT cells (AX4) under submerged conditions for 15 hours. The two lower panels show their development when deposited on Nitrocellulose filters and incubated for 15 and 36 hours, respectively. Scales bars: 40 μm (upper panels), 0.1 mm (middle panels) and 0.2 mm (lower panels).

The multicellular development of the different AX4 strains was studied in submerged development and on Nitrocellulose filters (Fig 7). The three strains that expressed *acaA* showed the same temporal pattern of development as AX4 cells. Cell streams were formed by 15 hours of submerged development (Fig 7, upper panels). In filter development, tipped aggregates were observed by 15 hours of development (Fig 7, middle panels) and fruiting bodies by 24 hours. However, the size of the aggregates and mounds of the strains expressing *acaA* from promoters 1 and 2 was smaller than that of WT and promoter 3-driven structures. Fruiting bodies were allowed to complete maturation (36 hours, Fig 7 lower panels) and the size of the sporocarpi determined. The diameter of the sori from the WT/Pr1::*acaA* strain was 37.2 ± 15% that of the WT ones. Similarly, the diameter of the WT/Pr2::*acaA* strain sori was 34.0 ± 12.3%, while the diameter of WT/Pr3::*acaA* sori was 75.0 ± 17.2% of the ones of the WT strain.

## Discussion

Expression of the *acaA* gene is regulated by three promoters, that are active at different developmental stages and/or regions of the developing structures [26]. In this article, the *acaA* gene was expressed under the control of each of these promoters in an *acaA*-mutant AX4 strain. The more distal promoter, Promoter 1, is active at the initial cell aggregation stage that is mediated by extracellular cAMP in *D*. *discoideum* [23]. Expression of *acaA* under control of this promoter recovered aggregation in the *acaA*-mutant strain, as evidenced by the morphological change of the cells and the formation of aggregates under submerged conditions and on Nitrocellulose filters, the expression of the *tgrC5* gene, that is dependent on extracellular cAMP signalling, and of prestalk and prespore genes. Basal expression of *carA* is not dependent on cAMP signalling [32] and the gene is expressed in the *acaA*-KO strain. However, expression of *acaA* under control of promoter 1 (and also promoters 2 and 3) increased *carA* mRNA levels in agreement with previous studies indicating that *carA* expression is regulated by cAMP [35, 38]. Expression of *acaA* under Promoter 1 produced aggregates smaller than wild-type cells and less tightly packed indicating that additional activation of *acaA* expression driven by promoters 2 and/or 3 could be required for a strong aggregation process. Post-aggregative development was poorly recovered by *acaA* expression driven by promoter 1 and only few, small, structures were formed. The prespore gene *pspA* and the prestalk gene *ecmA* were also expressed to much lower levels than in wild-type structures at 24 hours of development, indicating that very few cells differentiated into prespore or prestalk cells.

Expression of *acaA* under control of promoter 2 resulted in formation of tight aggregates and the expression of cell adhesion molecules, such as *tgrC5*, was induced. However, aggregates were smaller than wild-type ones and few, small, culminant structures were formed. In agreement with these data, the expression of *ecmA* and *pspA* was very low in the strains complemented from promoter 2. The developmental pattern of *acaA* expression shown in Fig 1 indicates that promoters 2 induced very high levels of *acaA* mRNA with maximal expression at 9 hours of development, as WT cells.

*acaA* expression driven by promoter 3 resulted in the more complete recovery of the developmental process. These data are in agreement with previous results showing that a smaller fragment of the promoter 3 region analyzed in this article [39] and a promoter 3 fragment fused to part of promoter 2 [40] recovered the development of *cbfA* and *acaA* mutants generated in the DH1 *D*. *discoideum* strain, respectively. Promoters 2 and 3 differed at the cell type where they are active, prespore cells in the case of promoter 2 and prestalk tip cells in the case of promoter 3. This difference could be important for their distinct biological activity since prestalk cells have been described as an important signalling centre during intermediary stages of *D*. *discoideum* development such as finger formation, slug migration and the initiation of culmination [41, 42]. Adenylyl cyclase A has been previously shown to play a relevant role in this signalling pathway [40]. Expression of *acaA* in prestalk tip cells could complement this signalling process in the mutant strain to produce a more robust developmental process and larger structures. These data would enforce the importance of *acaA* expression at the prestalk region.

*acaA* expression levels have an important role in developmental regulation since previous reports showed that *acaA* expression under control of a strong, constitutive actin promoter resulted in small structures [36, 37]. Over-expression of *acaA* could impair the aggregation process by reducing the size of the aggregation field. This could be the case when *acaA* is expressed from promoter 1 in WT cells since expression of this gene is induced precociously, at 3 hours of development, and at higher levels than the endogenous gene (Fig 6). It could be also the case for promoter 2-driven expression where the temporal pattern of expression is similar to that of the endogenous gene but expression levels are about 100 times higher (Fig 6). Over-expression driven by promoter 2 could also impair post-aggregative development since an inhibitory role of cAMP on stalk cell differentiation has been described [43, 44]. However, the structures formed by the *acaA*-KO strain expressing Pr1::*acaA* and Pr2::*acaA* were much smaller than those of the AX4 strain expressing these constructs. Therefore, *acaA* over-expression is not the only reason for the small size of the structures formed by the mutant strain which indicates that expression from promoters 1 and 2 do not completely complement the *acaA* expression required for post-aggregative development. In contrast, expression of *acaA* from promoter 3 in WT cells did not affect the developmental process significantly and only a discrete decrease in the size of the fruiting bodies was observed.

Although the biological activity of promoter 3 is consistent in WT and *acaA*-KO strains, significant differences in the pattern of activity during development were found. In addition, the activity of promoter 3 had been previously studied using a β-galactosidase expression vector in wild-type AX4 cells [26]. In these studies significant induction of promoter 3 was observed only after 10 hours of development although β-galactosidase activity was observed in mound and tipped mound structures. However, *acaA* expression driven from this promoter was observed at earlier developmental stages in the present study, specially in AX4 cells. The differences described could be due to several reasons. One of the variables that could affect these results is the gene being expressed in each experiment. For example, the differences observed on β-galactosidase and *acaA* mRNAs expression could be due to a feed-back activation of promoter 3 by cAMP at early developmental stages. According to this hypothesis, high cAMP levels would be obtained by *acaA* expression that could increase promoter 3 activity. Greater stability of the β-galactosidase mRNA than the *acaA* mRNA could also contribute to increase β-galactosidase mRNA levels at late developmental stages.

A second source of variation is the strain used in each study. As mentioned above, acaA expression from promoter 3 was induced earlier in WT than in *acaA*-KO cells. The difference could be explained if promoter 3 activity is regulated by extracellular signals that are present in the WT strain and not in the *acaA*-KO strain.

A third important factor that could affect promoter 3 activity is the genomic context. In the previous study mentioned above the patterns of expression of the *acaA* mRNAs transcribed downstream of each promoter were determined. There is a good correlation with the data obtained previously for mRNAs 1 and 2, transcribed downstream of promoters 1 and 2 and the *acaA* mRNA expression driven by these promoters. Promoter 1 drove maximal expression between 3 and 6 hours in AX4 cells (Fig 6) and *acaA* mRNA1 showed maximal expression between 4 and 6 hours [26]. Promoter 2 activity was maximal at 9 hours (fig 6) and mRNA2 showed maximal expression between 8 and 10 hours [26]. Promoter 3 activity and mRNA3 expression showed very different expression levels, however. Induction of mRNA3 was only observed after 10 hours of development [26] while promoter 3 activity was maximal at 6 hours of development in WT cells (Fig 6). One possible explanation for these results is that regulatory elements present in promoters 1 or 2 or in other regions of the genome could also control mRNA3 expression. These interactions could explain the different pattern of expression under control of the isolated promoter 3 region or by the endogenous gene regulatory elements. Regulatory interactions are also observed in the decreased expression of the endogenous *acaA* mRNA in wild type cells when *acaA* is expressed under control of promoters 1 and 3 (Fig 6). Altogether, these data could indicate that promoter 3 activity is regulated in response to both intracellular and extracellular signals in cooperation with regulatory elements present in other genomic regions. The characterization of these possible regulatory regions and the regulation of their activity during development will require further detailed studies.

## Conclusions

The data reported in this article highlight some of the roles played by the three *acaA* promoters during development. In particular, expression from the aggregation-specific promoter 1 recovered aggregation but the expression from the tip-specific promoter 3 resulted in the more complete recovery of post-aggregative development. Expression of *acaA* in WT cells from promoters 1 and 2 resulted in smaller developmental structures, probably due to increased cAMP signalling during the aggregation process. The results obtained also indicate a complex regulation of *acaA* promoter 3 activity during development.

## Supporting Information

S1 Fig Analysis of *acaA*-mutant colonies generated by homologous recombination. *D*. *discoideum* AX4 cells were transfected with a plasmid vector were two adjacent regions of the *acaA* gene coding region (black boxes at the lower panel of the figure) were separated by a Blasticidine-resistance cassette (BsR, White box). Transformed colonies were selected by culture in the presence of blasticidine. Resistant clones were isolated by culture on agar plates overloaded with *K*. *aerogens*. DNA was prepared from three independent clones and used for PCR amplification using oligonucleotides hybridizing to the blasticidine-resistant cassette (Bsr-2, S1 Table) and to the *acaA* gene coding region downstream of the fragment used for construction of the KO plasmid vector (acaA S17, S1 Table), indicated by arrows in the lower panel of the figure. The products obtained were analyzed on an agarose gel as shown on lines 1, 2 and 3 of the upper panel of the figure. The migration of molecular weight markers is shown on lane M and their size in base pairs indicated at the right of the picture. DNA from clones 1 and 3 allowed amplification of the expected fragment of 1150 bp indicating the incorporation of the blasticidine cassette inside the *acaA* gene by homologous recombination. Clone 2 also incorporated the plasmid construct, as shown by the resistance to blasticidine, but it was not incorporated into the *acaA* gene.
(PDF)

S1 Table Oligonucleotides. (PDF)
