# Supplementary material for: Biological Activity of the Alternative Promoters of the Dictyostelium discoideum Adenylyl Cyclase A Gene
Source: PLoS One. 2016 Feb 3;11(2):e0148533. doi: 10.1371/journal.pone.0148533 (PMC4739590; doi:10.1371/journal.pone.0148533)
Supplement: S1 Fig — D. discoideum AX4 cells were transfected with a plasmid vector were two adjacent regions of the acaA gene coding region (black boxes at the lower panel of the figure) were separated by a Blasticidine-resistance cassette (BsR, White box). Transformed colonies were selected by culture in the presence of blasticidine. Resistant clones were isolated by culture on agar plates overloaded with K. aerogens. DNA was prepared from three independent clones and used for PCR amplification using oligonucleotides hybridizing to the blasticidine-resistant cassette (Bsr-2, S1 Table) and to the acaA gene coding region downstream of the fragment used for construction of the KO plasmid vector (acaA S17, S1 Table), indicated by arrows in the lower panel of the figure. The products obtained were analyzed on an agarose gel as shown on lines 1, 2 and 3 of the upper panel of the figure. The migration of molecular weight markers is shown on lane M and their size in base pairs indicated at the right of the picture. DNA from clones 1 and 3 allowed amplification of the expected fragment of 1150 bp indicating the incorporation of the blasticidine cassette inside the acaA gene by homologous recombination. Clone 2 also incorporated the plasmid construct, as shown by the resistance to blasticidine, but it was not incorporated into the acaA gene. (PDF) [file pone.0148533.s001.pdf]

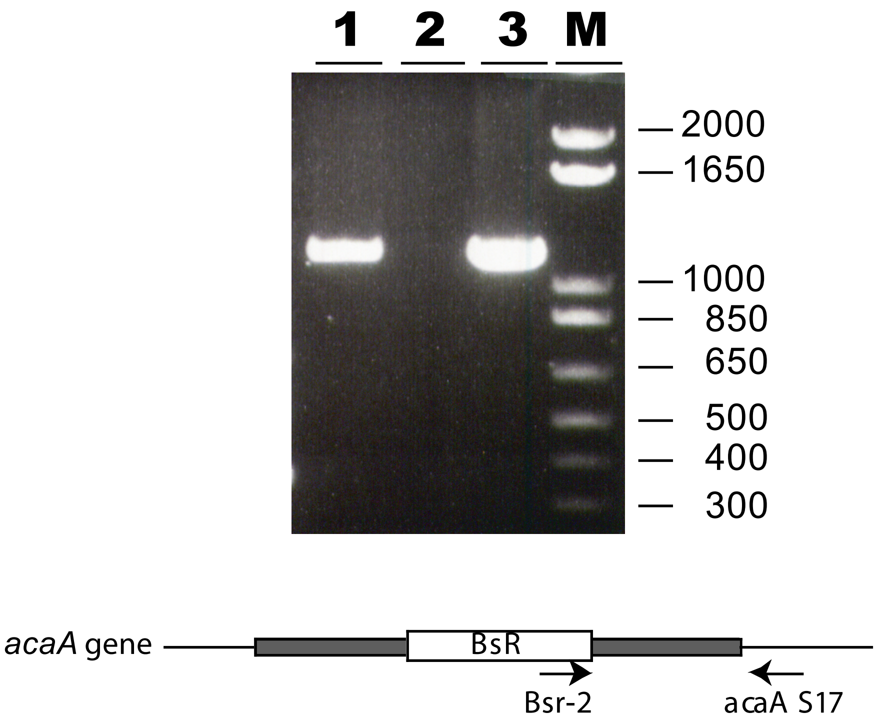

S1 Figure. Analysis of *acaA*-mutant colonies generated by homologous recombination.

D. discoideum AX4 cells were transfected with a plasmid vector were two adjacent regions of the *acaA* gene coding region (black boxes at the lower panel of the figure) were separated by a Blasticidine-resistance cassette (BsR, White box). Transformed colonies were selected by culture in the presence of blasticidine. Resistant clones were isolated by culture on agar plates overloaded with *K. aerogens*. DNA was prepared from three independent clones and used for PCR amplification using oligonucleotides hybridizing to the blasticidine-resistant cassette (Bsr-2, S1 Table) and to the *acaA* gene coding region downstream of the fragment used for construction of the KO plasmid vector (acaA S17, S1 Table), indicated by arrows in the lower panel of the figure. The products obtained were analyzed on an agarose gel as shown on lines 1, 2 and 3 of the upper panel of the figure. The migration of molecular weight markers is shown on lane M and their size in base pairs indicated at the right of the picture. DNA from clones 1 and 3 allowed amplification of the expected fragment of 1150 bp indicating the incorporation of the blasticidine cassette inside the *acaA* gene by homologous recombination. Clone 2 also incorporated the plasmid construct, as shown by the resistance to blasticidine, but it was not incorporated into the *acaA* gene.
