# Supplementary material for: Biological Activity of the Alternative Promoters of the Dictyostelium discoideum Adenylyl Cyclase A Gene
Source: PLoS One. 2016 Feb 3;11(2):e0148533. doi: 10.1371/journal.pone.0148533 (PMC4739590; doi:10.1371/journal.pone.0148533)
Supplement: S1 Table — (PDF) [file pone.0148533.s002.pdf]

S1 Table. Oligonucleotides

RT-qPCR; endogenous *acaA*	F- 5'- ccgcctctagaatgcaatcc-3'<br>R- 5'- gttgtgttttgccttcttaacttg-3'
RT-qPCR; plasmidic *acaA*	F- 5'- ccgcctctagaatgcaatcc-3'<br>R- 5'- gcttttgctccatagctttgg-3'
RT-qPCR; *carA*	F- 5'-atgatgataaagaagatgaagatgaacc-3'<br>R- 5'-ccagcactcaatattctcc-3'
RT-qPCR; *tgrC5*	F- 5'-gctggcttagcactttcatcag-3'<br>R- 5'-gagaccaacggcagcgacac-3'
RT-qPCR; *ecmA*	F- 5'-tgtgactgttcatcaggttgtc-3'<br>R- 5'-caaccagttgatggtgagca-3'
RT-qPCR; ; *pspA*	F- 5'-gtgcttcagtagatgtcatac-3'<br>R- 5'-ggtgttggtgttactgttggtg-3'
RT-qPCR; mtrRNA	F- 5'-gggtagtttgactggggcgg-3'<br>R- 5'-cactttaatgggtgaacacc-3'
acaA Promoter 1 cloning	F- 5'-ggtctagacttgatgagtggcccaaaacc-3'<br>R- 5'-ggctgcagatccaagaattcgtatctaaaaac-3'
acaA Promoter 2 cloning	F- 5'-ggtctagagtttttagatacgaattcttggatc-3'<br>R- 5'-ggctgcagcaaagatatatttatgaagtgagg-3'
acaA Promoter 3 cloning	F- 5'-ggtctagacctcacttcataaatatatctttg-3'<br>R- 5'-ggctgcagcttttttttttgtgattattattattattac-3'
acaA Open Reading Frame cloning	F- 5'-ggctgcagatggcatctagctcaccaatg-3'<br>R- 5'-ggggatccacttgaaagatggaatcttggag-3'
KO colonies analysis, Bsr-2	F- 5'-cagccaaagagtatgaaaatc-3'
KO colonies analysis, acaA S17	R- 5'-ctctctatagttcattattcatctttcc-3'
